# Distracted Driving on YouTube: Categorical and Quantitative Analyses of Messages Portrayed

**DOI:** 10.2196/14995

**Published:** 2020-02-10

**Authors:** Marko Gjorgjievski, Sheila Sprague, Harman Chaudhry, Lydia Ginsberg, Alick Wang, Mohit Bhandari, Bill Ristevski

**Affiliations:** 1 Centre for Evidence-Based Orthopaedics McMaster University Hamilton, ON Canada; 2 Division of Orthopaedic Surgery University of Toronto Toronto, ON Canada; 3 Division of Orthopaedic Surgery McMaster University Hamilton, ON Canada; 4 Department of Neurosurgery University of Ottawa Ottawa, ON Canada

**Keywords:** distracted driving, YouTube, texting and driving, car distractions, cell phones, orthopedic injuries

## Abstract

**Background:**

Distracted driving is a global epidemic, injuring and killing thousands of people every year. To better understand why people still engage in this dangerous behavior, we need to assess how the public gets informed about this issue. Knowing that many people use the internet as their primary source of initial research on topics of interest, we conducted an assessment of popular distracted driving videos found on YouTube.

**Objective:**

This study aimed to gauge the popularity of distracted driving videos and to assess the messages portrayed by classifying the content, context, and quality of the information available on YouTube.

**Methods:**

We conducted a search on YouTube using 5 different phrases related to distracted driving. Videos with more than 3000 views that mentioned or portrayed any aspect of distracted driving were identified, collected, and analyzed. We measured popularity by the number of videos uploaded annually and the number of views and reactions. Two independent researchers reviewed all the videos for categorical variables. Content variables included distractions; consequences; orthopedic injuries; and whether the videos were real accounts, reenactments, fictitious, funny, serious, and graphic. Context variables assessed the setting of the events in the video, and quality of information was measured by the presence of peer-reviewed studies and inclusion and referencing of statistics. Discrepancies in data collection were resolved by consensus via the coding authors. A comparative subanalysis of the 10 most viewed videos and the overall results was also done.

**Results:**

The study included a total of 788 videos for review, uploaded to YouTube from 2006 to 2018. An average of 61 videos with greater than 3000 views were uploaded each year (SD 34.6, range 3-113). All videos accumulated 223 million views, 104 million (46.50%) of them being among the 10 most viewed videos. The top 3 distractions depicted included texting, talking on the phone, and eating and/or drinking. Motor vehicle crashes (MVCs) and death were depicted in 742 (94.2%) videos, whereas 166 (21.1%) of the videos depicted injuries. Orthopedic injuries were described in 90 (11.4%) videos. Furthermore, 220 (27.9%) of the videos contained statistics, but only 27 (3.7%) videos referenced a peer-reviewed study.

**Conclusions:**

This study demonstrates that there is a high interest in viewing distracted driving videos, and the popularity of these videos appears to be relatively stable over time on a forum that fluxes based on the current opinions of its users. The videos mostly focused on phone-related distractions, overlooking many other equally or more common forms of distracted driving. Death, which in reality is a far less common distracted driving consequence than injuries, was portrayed 1.7 times as much. Surprisingly, orthopedic injuries, which lead to a massive source of long-term disability and often result from MVCs, are vastly underrepresented.

## Introduction

### Background

Distracted driving is a global epidemic and has become the number one killer of teenagers [1]. In North America alone, distracted driving plays a role in approximately 4 million road traffic accidents a year [2]. According to Mark Edwards, Director of Traffic Safety at the American Automobile Association, somewhere between 25% and 50% of all motor vehicle crashes (MVCs) in the United States are directly related to driver distraction as the root cause of automobile accidents [3]. In addition, injuries resulting from MVCs are in the top 10 causes of disability and are expected to climb to the top 3 by 2030 [4]. The World Health Organization estimates that in 2016, there were 1.35 million road traffic fatalities [5], and it recommended that future research focus on traffic injuries.

Data from the National Highway Traffic Safety Administration for 2015 suggested that for every traffic fatality that occurred because of distracted driving, approximately 113 people were injured (3477 fatalities to 391,000 accident-related injuries) [6]. Many of these acute injuries secondary to this type of high-energy trauma lead to permanent impairments and/or disabilities. In addition, MVCs in the United States are estimated to total US $40 billion in direct costs and US $123 billion in societal costs [6,7].

Distracted driving is anything that diverts a driver’s attention from safely operating a vehicle and subsequently reduces the driver’s awareness and driving ability, leading to a potential risk of compensating actions or crashing [8]. The diversion of attention should not be because of alcohol, drugs, fatigue, or a health condition [9,10]. Texting or talking on a mobile phone, daydreaming, eating and/or drinking, and using a navigation system while driving are just a few examples of distracted driving. Wickens’ Multiple Resource Theory (MRT) [11] explains driving as a visual-spatial-motor task, using cognitive, visual, and motor resources concurrently [12,13]. According to MRT, tasks that compete for the same resource can cause dual-task interference in any or all 3 resources, leading to decreased driving ability.

Cognitive distractions happen when a driver’s mind concentrates on mental tasks other than driving, for instance, daydreaming or talking on a hands-free mobile phone.

Visual distractions occur when a driver shifts their gaze away from the safe operation of the vehicle, such as looking at a map.

Manual distraction occurs when the driver takes one or both hands off the steering wheel for any reason. Some common examples include eating and drinking or adjusting the radio in the car.

An activity such as texting while driving is especially dangerous as it combines all 3 elements of distraction—cognitive, visual, and manual [14]. In fact, text messaging while driving may increase the relative risk of being involved in a collision 23 times [15-18]. The National Safety Council estimates that in 2013, 26% of crashes involved mobile phone use [15]. Although mobile phone use while driving may be the catalyst for renewed concern of distracted driving, some studies have estimated the use of mobile phone devices as the second most common distraction in fatal crashes with 14%, compared with daydreaming with 61% [6,19].

### Objectives

It is clear from the data that distracted driving is very dangerous and also very prevalent. Knowing that most people use the internet as their primary source of information on such topics, we directed our research focus on YouTube, the most popular video sharing platform on the internet [20]. A previous study has examined whether YouTube can be used as an educational platform for curbing adolescent cell phone use while driving [21]. However, no studies have assessed the general messages being portrayed in distracted driving videos and the most common elements in them, such as types of distractors portrayed, consequences of distracted driving, and statistics about distracted driving. Understanding this core information that is presented to viewers is novel and will be critical in mitigating the harms of distracted driving.

The specific goals of the study were to gauge the popularity and to categorize and assess the messages being delivered by distracted driving videos, by methodically classifying the content, context, and quality of the information available on YouTube.

## Methods

### Study Design

The *Distracted Driving on YouTube* study is a cross-sectional study examining popular distracted driving videos found on the video sharing platform YouTube. The following 5 different combinations of keywords and phrases were employed to search YouTube for distracted driving videos: “distracted driving”, “car distractions”, “cell phone and driving”, “drivers not paying attention”, and “texting and driving”. In the development of our search phrases, we relied on YouTube’s smart search, which autopopulates potential searches with the most common terms containing those words. Owing to public opinion, government campaigns, and the focus of distracted driving literature on mobile phone distractions, we also included phrases that covered these types of distractions. We conducted the search on a single day, June 13, 2018, and sorted the search results by view count.

### Screening and Collection of Videos

YouTube videos uploaded after 2006, with more than 3000 views, that discussed or demonstrated any aspect of distracted driving were collected and analyzed. We excluded from the study any videos that did not demonstrate or mention distracted driving, videos about reckless, careless, or impaired driving, and videos not in English. User channels that appeared in the search results and copies of collected videos were not included in our review. Identical videos appearing in the results of multiple search phrases were analyzed and coded only once.

### Data Acquisition and Analysis

The videos were examined by 2 independent reviewers, and any discrepancies in data collection were resolved by consensus via the coding authors. The overall interrater reliability measurement was calculated as the mean from all the kappa results for the examined variables, which were calculated using the Cohen kappa formula for 2 raters. The study recorded the popularity of the videos via quantitative variables, such as the date and year the video got uploaded and the number of views, likes, and dislikes it received. Videos from a different uploader, but with identical content, were labeled as duplicates and only contributed data for the quantitative variables. They were not included in the final analysis of the categorical variables.

We recorded categorical variables under the categories of content, context, and quality of information. Content variables included type (eg, phone, texting, talking on a phone, talking with a passenger, eating/drinking, and daydreaming) and form (eg, cognitive, visual, manual, or combinations) of the distraction; consequences (eg, crash, death, injury, and legal); orthopedic injuries specifically; and whether the videos were real accounts, reenactments, fictitious, funny, serious, and/or graphic. We considered a video to be graphic when there was an explicit or visual depiction of a serious injury or death. Context variables included whether the video was a public safety announcement (PSA), television show or newscast, advertisement, or an amateur video. Finally, the quality of information variables included whether the video contained statistics and/or studies, and whether the studies were peer reviewed and the statistics referenced. In addition, we conducted a subanalysis of the 10 most viewed videos and performed a comparison with the overall results.

## Results

The search methods generated 5,520,000 results for all 5 search phrases, out of which 987 videos met our eligibility criteria. Once we removed the identical videos appearing in multiple searches, a total of 788 videos uploaded to YouTube from 2006 to 2018 were included for review. An average of 61 videos with greater than 3000 views were uploaded each year (SD 34.6, range 3-113). Table 1 shows the number of videos uploaded per year matching the search and inclusion criteria.

**Table 1:** Number of YouTube videos uploaded per year matching search and inclusion criteria.

Years	Number of videos with >3000 views per year
2006	3
2007	17
2008	20
2009	51
2010	61
2011	93
2012	74
2013	87
2014	85
2015	93
2016	113
2017	63
2018	28

We performed an analysis of the videos using various categorical variables listed in Table 2. Our interrater reliability measurement showed substantial agreement between the reviewers (κappa=0.73). The review demonstrated that PSAs accounted for 37.3% (294/788) of the videos, and more than half of the videos (440/788, 55.8%) were a depiction of a real account. Furthermore, an evaluation of the video’s content revealed that the vast majority of the videos were not comedic/funny in nature and were coded as serious (643/788, 81.6%).

Cognitive distractions, depicted in 91.0% (717/788) of the videos, were the most common form of distraction recorded, whereas manual and visual distractions were depicted in 82.6% (651/788) and 81.6% (643/788) of the videos, respectively. Further review showed that 27.9% (220/788) of the videos contained statistics, and 10.0% (79/788) referenced the source of the statistics, while 3.4% (27/788) videos mentioned a peer-reviewed study. In addition, 21.1% (166/788) of the videos included some form of an injury, whereas orthopedic injuries specifically were depicted in 11.4% (90/788) of the videos.

**Table 2:** Videos depicting examined categorical variables.

Categorical variables	Videos depicting variables, n (%)
Public safety announcement	294 (37.3)
Television show or newscast	135 (17.1)
Amateur	272 (34.5)
Advertisement	68 (8.6)
Real account	440 (55.8)
Reenactment	12 (1.5)
Fictitious	219 (27.8)
Cognitive distraction	717 (91.0)
Visual distraction	643 (81.6)
Manual distraction	651 (82.6)
Funny	89 (11.3)
Serious	643 (81.6)
Graphic	65 (8.2)
Contains statistics	220 (27.9)
Statistics referenced	79 (10.0)
Study mentioned/discussed^a^	60 (7.6)
Peer-reviewed study^b^	27 (3.4)
Orthopedic injury	90 (11.4)
Injury	166 (21.1)

^a^A study about distracted driving was mentioned in the video.

^b^The mentioned study in the video was published in a peer-reviewed journal.

All included videos were reviewed for specific distractions. Table 3 demonstrates the number and percentage of videos that portrayed a particular type of distraction. Overall, the 2 most depicted distractions were mobile phone related, and included texting and talking. Texting while driving was the number one distracting activity present in 64.6% (509/788) of all videos. Phone conversations, both handheld and hands free, were calculated together and were depicted in 24.5% (193/788) of the videos. Eating and/or drinking and radio manipulation each occurred in 9.4% (74/788) of videos, whereas talking with a passenger was displayed in 9.1% (72/788) of the videos. Daydreaming was present in 2.0% (16/788) of all videos. It should be noted that many videos contained more than one form of distraction.

Table 4 illustrates the consequences depicted in the videos. The 3 most common outcomes were crash or an accident, seen in 58.4% (460/788) of the videos, followed by death and injury at 35.8% (282/788) and 21.1% (166/788), respectively. Similar to the specific distractions, multiple consequences were often shown in a single video.

**Table 3:** Videos containing a specific driving distraction.

Driving distractions	Value, n (%)
Texting (phone)	509 (64.6)
Talking (phone)	193 (24.5)
Eating/drinking	74 (9.4)
Radio	74 (9.4)
Talking with a passenger	72 (9.1)
Unknown^a^	67 (8.5)
Phone (app, Web, music, pic, video, reading)	59 (7.5)
In-vehicle distraction+reaching for an object	57 (7.2)
Grooming	55 (7.0)
Other^b^	51 (6.5)
Programming navigation/GPS systems	34 (4.3)
Outer-vehicle distraction	32 (4.1)
Interacting with children	24 (3.0)
Electronic device (laptop, computer, Mp3, iPod)	21 (2.7)
Reading	18 (2.3)
Daydreaming	16 (2.0)

^a^Distractions were not visible.

^b^Distractions were too few to categorize independently.

**Table 4:** Videos containing a specific consequence of distracted driving.

Consequences	Value, n (%)
Car crash/accident	460 (58.4)
Death	282 (35.8)
Injury	166 (21.1)
None	130 (16.5)
Orthopedic injuries	90 (11.4)
Fine (ticket)	82 (10.4)
Near crash/accident	32 (4.1)
Incarceration	27 (3.4)
Police pull over	23 (2.9)
Legal	13 (1.6)
Warning	9 (1.1)
Other	38 (4.8)

As the 10 most viewed videos accounted for nearly half of all the views garnered, a separate review of these videos was conducted. Table 5 represents the various distractions recorded in these videos. The use of mobile phones was once more the most common distracting activity, especially texting, which was present in all (10/10, 100%) videos.

Table 6 describes the consequences depicted in the 10 most viewed videos. A car crash or an accident was the most common consequence depicted in 70% (7/10) of the videos. Death was depicted in 60% (6/10) of videos, and injury was shown in 40% (4/10) of the videos.

**Table 5:** Specific distractions in the 10 most viewed videos.

Distractions	Value, n (%)
Texting (phone)	10 (100)
Talking with a passenger	4 (40)
Eating/drinking	3 (30)
Talking (phone)	1 (10)
Radio	1 (10)
Interacting with children	1 (10)
Other	1 (10)

**Table 6:** Specific consequences in the 10 most viewed videos.

Consequences	Value, n (%)
Car crash/accident	7 (70)
Death	6 (60)
Injury	4 (40)
Orthopedic injury	2 (20)
None	1 (10)

Table 7 presents a comparative analysis of all the categorical variables between the 10 most viewed videos and all the videos reviewed. In terms of context, the majority of the 10 most viewed videos were PSAs (8/10, 80%). However, the percentage of total videos that were PSAs was substantially lower at 37.3% (294/788). In addition, 60% (6/10) of the top 10 videos viewed depicted fictitious events, compared with 27.8% (219/788) for the combined data. The character of the content was more evenly distributed among the top 10 videos, with 60% (6/10) being serious, 40% (4/10) funny, and 30% (3/10) considered as graphic, as opposed to all the videos, where the vast majority were serious in nature (643/788, 81.6%).

Our comparative analysis also included quantitative variables, which are displayed in Table 8. There were more than 223 million views distributed across all 788 examined videos (mean 17,211,395.2, SD 16,838,381.6), and 104 million (46.50%) of those views belonged to the 10 most popular videos. In addition, 48.94% (545,628/1,114,680) of all the reactions (likes/dislikes) and 50.76% of all likes (535,600/1,055,070) were among the top 10 videos.

**Table 7:** Videos depicting examined categorical variables in the 10 most viewed videos and all videos.

Categorical variables	Ten most viewed videos depicting variable, n (%)	All videos depicting variable (N=788), n (%)
Public safety announcement	8 (80)	294 (37.3)
Television show or newscast	2 (20)	135 (17.1)
Amateur	0 (0)	272 (34.5)
Advertisement	2 (20)	68 (8.6)
Real account	3 (30)	437 (55.8)
Reenactment	0 (0)	12 (1.5)
Fictitious	6 (60)	219 (27.8)
Cognitive distraction	10 (100)	717 (91.0)
Visual distraction	10 (100)	643 (81.6)
Manual distraction	9 (90)	651 (82.6)
Funny	4 (40)	89 (11.3)
Serious	6 (60)	643 (81.6)
Graphic	3 (30)	65 (8.2)
Contains statistics	1 (10)	220 (27.9)
Statistics referenced	0 (0)	79 (10.0)
Study mentioned/discussed^a^	0 (0)	60 (7.6)
Peer-reviewed study^b^	0 (0)	27 (3.4)
Orthopedic injury	2 (20)	90 (11.4)
Injury	4 (40)	166 (21.1)

^a^A study about distracted driving was mentioned in the video.

^b^The mentioned study in the video was published in a peer-reviewed journal.

**Table 8:** Videos depicting examined quantitative variables in the 10 most viewed videos and all videos.

Quantitative variables	Ten most viewed videos	All reviewed videos
Views, n (%)	104,057,183 (46.50)	223,748,138 (100.00)
Likes, n (%)	535,600 (50.76)	1,055,070 (100.00)
Dislikes, n (%)	10,028 (16.82)	59,610 (100.00)
Total reactions, n (%)	545,628 (48.94)	1,114,680 (100.00)
Ratio of likes/dislikes	53.4	17.7

## Discussion

### Principal Findings

In this study, the 788 YouTube videos on distracted driving had more than 223 million combined views distributed across all years, demonstrating that there is a high interest in this topic. The number of videos with 3000 views or more uploaded per year was also relatively stable throughout the years. The increase in the number of uploaded videos after 2006 follows with YouTube inception and growth in popularity. In addition to the fact that 2018 was truncated in June, and represents approximately 6 months of videos rather than a full year, the decline in 2017 to 2018 also has to be viewed in the context that the newest videos have the least amount of time to garner views (and will gain views with additional time).

A large proportion of the videos were PSAs (294/788, 37.3%), with more than one-third of the videos (272/788, 34.5%) having amateur content. Videos containing statistics with a referenced source were 10.0% (79/788), and only 3.4% (27/788) videos quoted peer-reviewed studies, signifying most videos were opinion based. Therefore, the videos demonstrated a significant disparity between the information presented on this forum and the current data available on distracted driving. For instance, the results showed texting as the most commonly observed distracting activity (509/788. 64.6%). However, using data from the Fatality Analysis Reporting System, Erie Insurance reports that among the top 10 distractions involved in fatal car crashes, mobile phone use ranked as second with 14%, behind *lost in thought* or daydreaming with 61% [6,19]. In this study, daydreaming was the least represented type of distraction. This discrepancy is in alignment with data that demonstrate that the majority of people believe that mobile phones are the number one distraction when driving [22]. It should be noted that commonality is not to be mistaken for the magnitude of distraction that can occur. Studies have placed programming navigation/GPS systems and texting while driving as the most distracting tasks [23,24]. In this study, programming a navigations/GPS system was seen in only 4.3% (34/788) of all videos, whereas texting while driving was depicted in 64.6% (509/788) of the videos. Both tasks are obviously exceedingly dangerous, but it is interesting to see that mobile phones are dominating this public forum, although some of the more common and potentially equally dangerous distractions identified garner so little spotlight.

In the videos, death as a result of distracted driving is grossly overrepresented (282/788, 35.8%) relative to injuries sustained in the same circumstances (166/788, 21.1%). Information available from reported MVCs suggests that injuries from distracted driving crashes are nearly 113 times more likely than fatalities [6]. Furthermore, according to 2011 Canadian data from the National Trauma Registry [25], the most common cause of major injury were MVCs, with 79% of these people having sustained musculoskeletal injuries. However, in this study, 21.1% (166/788) of the videos depicted some form of injury, whereas orthopedic injuries were depicted in only 11.4% (90/788) of the videos, representing once again a huge disparity from available data on distracted driving.

A possible reason for the dominance of serious outcomes such as death is the uploader’s goal of reaching and engaging people for more views, reactions, and comments or demonstrating the extremes to get viewers to think about distracted driving. Although we hope this may build awareness around the risks of distracted driving, it presents messages that can certainly be dramatically different from reality, for example, directing one’s attention to death as a consequence of distracted driving but massively underrepresenting a life-altering injury with permanent impairment and/or disability.

Furthermore, because of the fact that the 10 most viewed videos garnered 46.5% (104,057,183/223,748,138) of all the views and more than half of all likes (535,600/1,055,070, 50.76%), we performed a subanalysis of these videos and found similar results. Car crashes and death were the 2 most common outcomes. Mobile phone use, particularly texting, was present in all 10 videos. Interestingly, there were no studies or statistics referenced in the 10 most viewed videos.

### Limitations

This study had several limitations. Data gathering for categorical variables relied on the investigator’s ability to scan and detect for the variables, allowing for the possibility to overlook some information. Furthermore, there are no standardized methods for analyzing YouTube videos; thus, the interpretation of various variables depended on the researcher’s judgment, which could lead to bias. We minimized these issues by assigning 2 independent reviewers to analyze each video and resolved any discrepancy between the coded data by consensus via the coding authors. To our knowledge, this is the largest YouTube study on distracted driving, and the large sample size would decrease the potential data skewing that can be observed with smaller sample sizes.

### Comparison With Prior Work

YouTube is the second most visited website in the world, with 5 billion videos and 1 billion hours of content viewed daily, and it is by far the most used video sharing platform on the internet [20,26]. It is available in 76 languages in 88 countries, which is 95% of all internet users [20], and offers a unique opportunity to reach an audience of millions. Other studies have investigated the sharing potential of YouTube for information on various medical issues, such as immunizations [27], concussions [28], heart transplantation [29], and sedentary behaviors [30]. A recent study about videos on distracted driving on YouTube in 2017 analyzed 100 videos specifically on mobile phone use as it applies to adolescents [21]. However, they only focused on 1 form of distraction only in adolescents, compared with this study, which examined multiple forms and types of distractions and the messages portrayed in them.

### Conclusions

This study demonstrates the overall messages portrayed in videos on YouTube focused on distracted driving and shows discrepancies between current data on distracted driving and what is described.

The popularity of viewing videos on this topic appears to be high and relatively stable over time on a forum that fluxes based on the current opinions of its users. This is encouraging in the sense that people are being exposed to the dangers of distracted driving. However, overall information presented in these videos can mostly be classified as opinion based, with a paucity of referenced statistics or data from peer-reviewed studies.

Videos most often focused on texting while driving and the most dramatic consequences such as MVCs and death. Although we hope this brings attention to the seriousness of distracted driving, it is not representative of the known data on distracted driving. In studies, the most demanding task while driving is potentially programming a navigation/GPS system and/or texting while driving [23,24], whereas the most common distraction is thought to be daydreaming [19]. Unfortunately, daydreaming and programming a navigation/GPS system are largely ignored in these videos and represent critical information to know about distracted driving. In addition, death was portrayed more than 1.7 times compared with injury in terms of potential consequences of distracted driving. In reality, injuries are 113-fold more common compared with fatalities [6]. Similarly, injuries in general and specifically orthopedic injuries, which are exceedingly common and can lead to a massive source of long-term disability and/or impairment, are vastly underrepresented compared with reality.

Future research may be aimed at potentially harnessing this interest on YouTube with respect to distracted driving to ascertain whether perspectives and behaviors can be favorably altered to minimize distracted driving.

## Abbreviations

MRT: Multiple Resource Theory
MVC: motor vehicle crash
PSA: public safety announcement
